# Case of an Exfoliation of the Anterior Part of the Upper Jaw Bone

**Published:** 1788

**Authors:** William Loftie

**Affiliations:** Surgeon at Canterbury


### VI. *Case of an Exfoliation of the anterior Part of the Upper Jaw Bone. Communicated in a Letter to Dr.* Simmons *by Mr.* William Loftie, *Surgeon at Canterbury.*

E. S. about twenty years ſince, when in London, had a tooth drawn; and, in extracting it, a conſiderable degree of violence was uſed. From that time ſhe perceived a tumour on the maxilla ſuperior, which gradually increaſed to the ſize of a pigeon's egg, acquired (as ſhe expreſſed herſelf) a ſtony hardneſs, and, on her taking cold, was attended with pain.

For this complaint ſhe occaſionally conſulted different ſurgeons, all of whom adviſed her not to meddle with it, but to leave it to nature.

About the beginning of the year 1780 the tumour increaſed, and was attended with more pain. A conſiderable inflammation now ſpread over the whole cheek, and towards the end of March ſhe perceived a fœtid purulent diſcharge from the ſide of the ſecond of the molares. An emollient fomentation was applied externally, and ſhe was directed to waſh her mouth frequently with warm milk. As I ſuſpected, from the diſcharge at the ſide of the tooth, that matter might be formed in the antrum maxillare, I perfuaded her to let me extract the tooth, in hopes of giving a free difcharge to the matter. This was done on the 5th of April, but without the wifhed-for effect. The difcharge ftill continued through the gum; and frequently fmall pieces of a dark-coloured, clayifh fubftance came with it. The fame applications were continued; and, as fhe complained of a conftant return of fever in the night, the bark was given.

She continued nearly in the fame ftate till about the 24th of May, when, on catching cold, the whole fide of the face was violently inflamed, and became very painful. It had an eryfipelatous appearance, and was attended with a good deal of fever. The bark was now omitted, and the emollient applications continued.

On the 30th of May the tumour extended from the nofe to the os temporis. The pain was very fevere, and the eye on that fide perfectly clofed. As the appearances now led me to apprehend great danger, I defired the affiftance of another furgeon. Two days afterwards a fluctuation was perceived, and on the 3d of June I made an opening on the moft prominent part of the tumour, which was near the external canthus of the eye. The difcharge was large, and extremely fœtid, and through this aperture came large pieces of the ſame clayiſh matter that was diſcharged through the gum. The maxilla ſuperior was found bare, and the probe could be paſſed obliquely downwards to the noſe. As a depending opening was to be wiſhed for, an inciſion was made at this part, and a ſeton introduced, which we hoped might promote the diſcharge downwards, and aſſiſt, by its friction, in ſeparating the diſeaſed bone. Upon examination through this lower aperture, the bone was found bare from its connexion with the alveolar proceſs to the os naſi, and from thence along the lower part of the orbit to the os malæ. The ſeton ſeeming to be of little uſe, it was left out, and the lower aperture was dilated upwards towards the junction of the os maxillare with the os naſi. This laſt inciſion gave great relief, but the diſcharge was ſtill conſiderable. The bark was now given freely, and an opiate adminiſtered occaſionally. In a few days the bone appeared looſe, and a ſmall piece came through the upper opening; and on the 7th of July I extracted the remainder, which appeared to be the whole of the anterior part of the maxilla ſuperior, from its connexion with the os naſi, extending along the orbit of the eye to the os malæ, and from thence, by the alveolar procefs, almoſt to the alæ naſi. The length of the extracted bone, from the os naſi to the os malæ, was two inches and a quarter, and its breadth near one inch and a quarter.

The removal of the bone was followed by a compact, putrid, dark-coloured maſs, reſembling clay, as before mentioned; and a ſubſtance of the ſame kind was found poſſeſſing the whole concave part of the maxilla joining to the naſal bone.

From this time the pain ceaſed, and the diſcharge gradually leſſened; and before the end of July the opening near the external canthus was healed, and the tumour of the cheek had almoſt wholly ſubſided. A ſmall diſcharge, however, ſtill continued through the inciſion near the os naſi, and at times a little purulent matter came through the gum: but in a ſhort time the wounds cloſed, and the patient has ever ſince remained perfectly well.

*Canterbury*,

Dec. 24th, 1787.

